# Risk of retinal vein occlusions in patients taking systemic tyrosine kinase inhibitors

**DOI:** 10.1038/s41433-026-04240-7

**Published:** 2026-02-06

**Authors:** Nitesh Mohan, Sunil K. Srivastava, Chandni Duphare, Timothy D. Gilligan, Mir Yusuf Ali, Moshe C. Ornstein, Ronald Sobecks, Dale Shepard, Sumit Sharma

**Affiliations:** 1 Cole Eye Institute, Cleveland Clinic, Cleveland, OH USA; 2 Cleveland Clinic Lerner College of Medicine of Case Western Reserve University, Cleveland, OH USA; 3 Department of Hematology and Medical Oncology, Cleveland Clinic Taussig Cancer Institute, Cleveland, OH USA

**Keywords:** Retinal diseases, Risk factors

## Abstract

**Objective:**

To describe a series of patients who developed retinal vein occlusion (RVO) while undergoing treatment with tyrosine kinase inhibitors (TKIs) for systemic malignancy.

**Methods:**

A retrospective chart review was performed to identify patients at an academic medical centre from 2014 to 2024 who developed an RVO while on TKI therapy. Data collected included demographics, cancer diagnosis, TKI agent and treatment duration, ocular history, and treatment outcomes. Ophthalmic imaging obtained at the time of presentation was reviewed when available to confirm the diagnosis of an RVO.

**Results:**

Eleven patients (12 eyes) were identified with an RVO during TKI therapy. The mean age at presentation was 75.9 ± 9.8 years, and 8 patients (72.7%) were male. TKIs included imatinib (*n* = 3), axitinib (*n* = 5), ibrutinib (*n* = 2), and regorafenib (*n* = 1). RVO developed after a mean duration of 2.8 ± 2.0 years on TKI therapy (range: 0.8–6.5 years). Of the 12 RVOs, 8 were central retinal vein occlusions (CRVOs) and 4 were branch retinal vein occlusions (BRVOs). The mean Naranjo Adverse Drug Reaction Probability Score was 5.2, suggesting a probable link between TKI use and RVO. One patient developed bilateral RVO after continuing regorafenib therapy.

**Conclusions:**

This series highlights a possible association between TKI therapy and RVO, underscoring the need for awareness in patients with vascular risk factors.

## Introduction

Tyrosine kinase inhibitors (TKIs) have revolutionised the field of oncology, offering targeted and effective cancer therapy with limited systemic side effects compared to traditional chemotherapy [1, 2]. TKIs inhibit tyrosine kinase activity to interfere with the aberrant signalling pathways driving cancer growth [2]. TKIs can be divided into subclasses based on the specific growth factor pathway that is inhibited, including Epidermal Growth Factor Receptor (EGFR) inhibitors, Breakpoint Cluster Region-Abelson (BCR-ABL) inhibitors, and Vascular Endothelial Growth Factor Receptor (VEGF-R) inhibitors [1]. The specific TKI chosen for a patient’s cancer is dependent on what growth factor/pathway is dysregulated [3, 4]. Due to the targeted nature of each TKI agent, it is thought to be less toxic to normal tissue. However, various side effects have been reported with TKI use, including ocular adverse effects such as keratitis, macular oedema, and periorbital oedema [5]. There have also been individual case reports of retinal vein occlusion (RVO) following TKI use [6–12].

RVOs are vascular occlusions of the retinal veins that can lead to significant complications that can cause vision loss, including macular oedema, neovascular glaucoma, proliferative retinopathy, and vitreous haemorrhage [13]. Although the exact aetiology of RVO is not known, common risk factors include hypertension, diabetes, hyperlipidaemia, and a hypercoagulable state [13]. Certain systemic medications have also been identified as a risk factor for RVO, with TKIs recently emerging as a potential contributor. Case reports have documented RVO development after use of certain TKIs including axitinib, regorafenib, sorafenib, lenvatinib, anlotinib, and ibrutinib [6–12, 14]. There have been limited reports showing multiple patients who have developed an RVO following TKI use, making it difficult to assess whether there is a true association. We present a single institution consecutive case series reporting 12 eyes (11 patients) that had RVO development following the use of various TKI agents.

## Methods

**Fig. 1:**
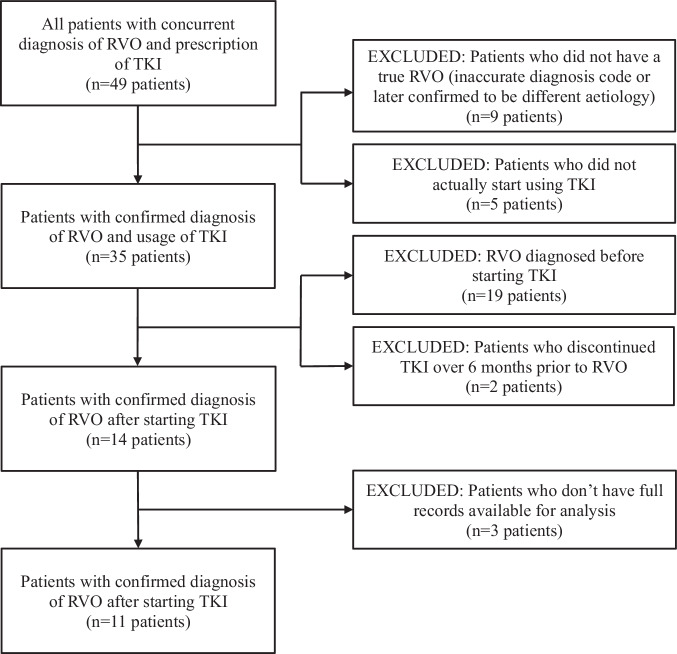
Diagram showing number of excluded patients from initial search and reason for exclusion.

This study was approved by the Institutional Review Board of the Cleveland Clinic Foundation and complied with the tenets of the Declaration of Helsinki. The requirement for informed consent was waived by the Institutional Review Board due to the study’s retrospective nature. A retrospective review of the electronic medical record was conducted for all patients at the Cleveland Clinic Cole Eye Institute who had use of a tyrosine kinase inhibitor and a diagnosis of a retinal vein occlusion (RVO) from January 2014 to January 2024. Initial search consisted of patients who had concurrent diagnosis of an RVO and prescription for a TKI, which yielded 49 patients. Further chart review was performed to exclude patients who never started their TKI, those who did not have temporal correlation between start of TKI and RVO, and those without a confirmed RVO diagnosis. Details of patients who were excluded from the initial search are detailed in Fig. 1. All RVOs were initially diagnosed by a retinal specialist based on dilated fundus exam and ophthalmic imaging (fundus photography, fluorescein angiography and optical coherence tomography (OCT)). For the purpose of this study, ophthalmic imaging on day of presentation was re-examined when available to confirm presence of RVO. Twelve eyes of eleven patients were identified and are reported here. Demographic data were collected for all patients. Individual chart review was performed to collect clinical data such as indication for TKI treatment, type of agent used, and visual acuity. For the purposes of this study, “held” was used to indicate a temporary interruption of TKI therapy, whereas “stopped” or “discontinued” indicated permanent discontinuation. Information on risk factors for RVO were also collected, including blood pressure, HbA1c, smoking status, and cholesterol levels. Likelihood of the RVO being related to TKI use was quantified using the Naranjo Adverse Drug Reaction Scale [15].

## Results

We reviewed 12 eyes (11 patients) who developed an RVO while on TKI therapy. Table 1 details both demographic and baseline clinical information for each eye. Average age at RVO presentation was 75.9 ± 9.8 years and 8 (72.7%) patients were male. All 11 patients had a diagnosis of pre-existing hypertension, 6 (54.5%) had diabetes mellitus, and 8 (72.7%) had a diagnosis of hyperlipidaemia. No patients in our cohort smoked at the time of their RVO, but 6 (54.5%) were former smokers. Five patients (45.5%) had chronic kidney disease, and 1 patient (9.1%) was diagnosed with heart failure. Outside of these factors and the patients’ actual malignancy, no other causes of thrombophilia were identified. Many patients also exhibited pre-existing ocular pathology. Six patients (54.5%) had non-proliferative diabetic retinopathy (NPDR) in both eyes (OU), 2 patients (18.2%) had primary open angle glaucoma (OU), and 2 patients (18.2%) had hypertensive retinopathy. For the 3 patients that had no relevant ocular history, imaging findings were re-examined to confirm lack of pathology. Initial indications for starting TKI therapy varied. Three (27.3%) patients were on TKI for chronic myeloid leukaemia (CML), 5 (45.5%) for renal cell carcinoma, 2 (18.2%) for chronic lymphocytic leukaemia (CLL), and 1 (9.1%) for gastrointestinal stromal tumour (GIST). Three patients were on imatinib, five patients on axitinib, two on ibrutinib, and one patient on regorafenib. Table 1 shows detailed information on each patient. Among the 11 patients, only 3 had prior TKI exposure. One patient had previously been on crizotinib, one had been on imatinib and sunitinib, and one had been on pazopanib.

**Table 1:** Demographic information for each patient in this reported series.

Patient	Eye	Sex	Age	TKI Used	TKI start date	Indication for TKI	Smoking Status	BP prior to RVO	HbA1c prior to RVO	LDL/HDL prior to RVO	Treatment for hypertension/diabetes/ hyperlipidaemia
1	OD	Male	80	Imatinib	May 2019	Chronic Myeloid Leukaemia	Former	138/78	6.0	48/40	None
2	OD	Male	59	Axitinib	September 2020	Renal Cell Carcinoma	None	115/60	5.3	82/57	Nifedipine
3	OD	Male	85	Ibrutinib	February 2012	Chronic Lymphocytic Leukaemia	Former	177/83	6.0	100/24	Metoprolol
4	OD	Male	84	Imatinib	April 2005	Chronic Myeloid Leukaemia	Former	161/86	4.9	47/38	Insulin glargine, Insulin aspart, Dulaglutide
5	OS	Male	72	Ibrutinib	May 2017	Chronic Lymphocytic Leukaemia	None	104/60	6.2	187/42	Amlodipine
6	OS	Male	93	Imatinib	March 2015	Chronic Myeloid Leukaemia	Former	136/71	6.2	96/25	Sacubitril/Valsartan, Carvedilol
7	OD	Female	79	Axitinib	February 2016	Renal Cell Carcinoma	None	131/66	5.6	106/61	Metoprolol, Atorvastatin
8	OD	Male	63	Axitinib	January 2013	Renal Cell Carcinoma	None	140/89	5.6	57/38	Lisinopril
9	OD	Female	73	Regorafenib	November 2021	Gastrointestinal Stromal Tumour	None	136/68	7.2	130/50	Carvedilol, Sacubitril/Valsartan Metformin, Insulin glargine, Insulin aspart,
	OS										
10	OD	Female	71	Axitinib	March 2020	Renal Cell Carcinoma	Former	122/66	6.4	53/60	Chlorthalidone, Losartan, Insulin, Atorvastatin
11	OS	Male	75	Axitinib	November 2017	Renal Cell Carcinoma	Former	148/63	4.8	33/36	Lisinopril, Atorvastatin

Cardiovascular risk factors such as blood pressure, HbA1c, and cholesterol levels were assessed at the most recent visit just prior to the presentation of the RVO.

Among the 12 eyes in our cohort, an RVO occurred after an average of 2.8 ± 2.0 years after initiating TKI therapy (range: 0.8 – 6.5 years). Full clinical details of each RVO are reported in Table 2. The presenting visual acuity (VA) ranged from 20/30 to count fingers (CF). Eight eyes were diagnosed with a central retinal vein occlusion (CRVO), while 4 eyes were diagnosed with a branch retinal vein occlusion (BRVO). Ten eyes were treated with an anti-VEGF agent (3 received only bevacizumab, 5 received both bevacizumab and aflibercept, and 2 received bevacizumab and a dexamethasone intravitreal implant). Two eyes (patient 4 and 11) did not receive treatment. VA at final follow-up ranged from 20/25 to CF. Six (50%) eyes showed improvement in VA from initial presentation, 3 (25%) stayed the same, and 3 (25%) worsened. The average follow-up time after experiencing an RVO was 3.5 ± 4.0 years.

**Table 2:** Information regarding each RVO presentation is also included from initial presentation to ultimate outcome of treatment.

Patient	Eye	Date of RVO	Months from starting TKI	TKI used (daily dosage)	Type of RVO	Disease in remission at time of RVO?	Presenting VA	Treatment	Final VA	Naranjo Score	Currently taking TKI?
1	OD	May 2022	36	Imatinib 400 mg	BRVO	Yes	20/250	Bevacizumab, Aflibercept	20/50	4	Yes
2	OD	March 2023	30	Axitinib 6 mg	BRVO	No	20/250	Bevacizumab	20/250	5	Held for six months due to decreased kidney function
3	OD	December 2015	44	Ibrutinib 520 mg	CRVO	Yes	CF	Bevacizumab, Aflibercept, Panretinal Photocoagulation	CF	5	Stopped due to RVO
4	OD	October 2006	18	Imatinib 400 mg	CRVO	Yes	20/400	None	20/200	3	Stopped due to improved/stable disease
5	OS	July 2022	60	Ibrutinib 420 mg	BRVO	Yes	20/200	Bevacizumab	20/80	5	Yes
6	OS	October 2021	78	Imatinib 133 mg	BRVO	Yes	20/40	Bevacizumab, Aflibercept	20/40	4	Yes
7	OD	June 2022	72	Axitinib 8 mg	CRVO	Yes	20/30	Bevacizumab	20/25	5	Held for one month due to RVO
8	OD	July 2014	18	Axitinib 10 mg	CRVO	Yes	20/30	Bevacizumab, Aflibercept	20/50	5	Stopped due to poor efficacy
9	OD	November 2022	12	Regorafenib 160 mg	CRVO	No	CF	Bevacizumab, Dexamethasone	20/150	8	Stopped due to second RVO
	OS	June 2023	19	Regorafenib 120 mg	CRVO	No	20/100	Bevacizumab, Dexamethasone	20/150	8	
10	OD	March 2021	12	Axitinib 6 mg	CRVO	Yes	20/80	Bevacizumab, Aflibercept, Faricimab	20/50	5	Stopped due to fatigue, nausea and diarrhoea
11	OS	September 2018	10	Axitinib 7 mg	CRVO	No	20/125	None	20/300	5	Stopped due to fatigue, nausea and diarrhoea

If the TKI is noted to be “Held,” it indicates a temporary interruption of therapy, while “Stopped” indicates permanent discontinuation of the TKI.

Total Naranjo scores can range from -4 to +13 with higher scores indicating that the observed event is more likely to be associated with the medication use [15]. The mean Naranjo score in our cohort was 5.2 (range: 3–8). The eye that received a score of 3 received points for the adverse event appearing after the suspected drug was given (+2), for the reaction not reappearing when given placebo (+1), and for the adverse event being confirmed by objective evidence (+1). That eye lost a point because there are alternative causes that could explain the RVO (-1). In addition to the aforementioned criteria, eyes that received a score of 4 additionally received a point for the adverse event improving when the drug was discontinued, or a specific antagonist was administered (+1). Eyes that received a score of 5 received an additional point due to the presence of previous reports of RVO related to the specific TKI that the patient used (+1). Lastly, the eyes that received a score of 8 received additional points for the adverse event reappearing after the drug was readministered (+2) and for the patient having a similar reaction to the same drugs in a previous exposure (+1), as she experienced a second RVO while on the TKI.

Three patients discontinued/held their TKI due to the RVO and did so immediately after the event. Five patients discontinued/held the TKI due to poor efficacy, systemic side effects, or stable/improved disease state. After the RVO, each patient in this group continued on their TKI for varying additional time before discontinuing the medication (Each had 13.2 years, 3.2 years, 1 month, 1 month, and <1 month follow-up, respectively). Three patients continued the same TKIs despite having a RVO and had an average 2.0 ± 1.4 years of additional follow up time. Among these 3 patients, only Patient 1 had their dose reduced after the RVO (due to other systemic side effects), while the rest continued their same dosage. These patients did not have worsening of symptoms in the affected eye or develop RVO in the contralateral eye.

## Case of bilateral CRVO

**Fig. 2:**
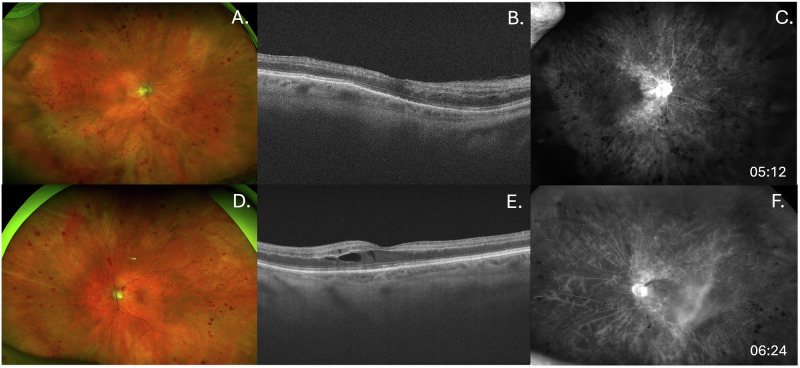
Imaging for Patient 9 upon presentation. Row 1 depicts imaging of the right eye on presentation of the first RVO with **A** diffuse haemorrhages on fundus photo, **B** extrafoveal intraretinal fluid with partial ellipsoid zone loss on OCT, and **C** diffuse leakage with significant nonperfusion on fluorescein angiogram. Row 2 depicts imaging of the left eye on presentation of the second RVO. **D** Fundus photo shows diffuse retinal haemorrhages, **E** OCT shows intraretinal fluid, and **F** fluorescein angiogram shows diffuse leakage with superonasal nonperfusion.

Of all the patients in our series, only one patient developed a bilateral RVO. Patient #9, a 73-year-old female with type 2 diabetes, hypertension, and chronic anaemia, was treated for GIST with imatinib, sunitinib, and regorafenib. In November 2022, approximately one year after starting regorafenib, she experienced sudden vision loss in her right eye (Presentation VA: CF; Previous VA: 20/20) and was diagnosed with a CRVO. Imaging demonstrated retinal non-perfusion and intraretinal fluid (Fig. 2A–C). Without initial treatment, intraretinal fluid decreased and OCT thickness improved. In June 2023, she developed a CRVO in the left eye (Presentation VA: 20/100; Previous VA: 20/30; Fig. 2D–F). Oral prednisone was initiated, but vision did not improve. She discontinued the regorafenib due to suspicion for the medication causing or increasing her risk for RVOs and started therapy with ripretinib. She received monthly bevacizumab injections (OU) and later intraocular dexamethasone implants (OU). As of February 2024, her vision remains stable at 20/150 (OU)

## Discussion

RVOs are potentially vision threatening complications associated with various systemic conditions. In this case series, we present 12 eyes from 11 patients who developed RVOs while receiving TKI therapy for their underlying malignancies. The occurrence of RVO in these patients raises important considerations regarding the potential association between TKIs and ocular adverse events. If TKI use is truly associated with development of RVOs, this adverse effect should be considered in planning treatment for patients and close follow-up with a retina specialist should be strongly encouraged for especially high-risk patients. Our series corresponds with previous reports which have shown RVOs while on axitinib, regorafenib, sorafenib, levatinib, anlotinib, and ibrutinib [6–12, 14]. While prior reports have documented individual events or detail a small series, our study provides a larger consolidated cohort with standardised clinical data and imaging, offering a more comprehensive characterisation of presentation, drug course, and outcomes.

Over the study’s inclusion period (January 1, 2014, to January 1, 2024), data extracted from the electronic health record identified 11,538 patients as having been prescribed a TKI at our tertiary care centre. This would represent a crude observed frequency of approximately 0.1%. However, this is likely an underestimate, as the actual number of patients who initiated a TKI is likely much lower. We were unable to determine how many patients filled or took the medication, and duration of use was not accounted for in this calculation. In addition, some patients who experienced an RVO while on TKIs may have followed with an ophthalmologist outside of our institution or had never underwent ophthalmic evaluation, which is not accounted for. Thus, the overall rate of RVO related to TKI is likely higher than 0.1%. In addition, although this rate suggests that the complication is relatively rare, it is a critical complication to consider, as it can result in lasting vision loss.

TKIs, especially those that target VEGF signalling, have been associated with an increased risk of thromboembolic events. Various analyses of clinical trial data have shown a significant increase in arterial thromboembolic events, but not necessarily in venous thromboembolic events [16–20]. It is unclear through what mechanism TKIs can increase risk of RVOs. However, it could be linked to alterations in cell signalling pathways that target cells in the eye and subsequently induce a hypercoagulable state. For example, certain TKIs that target the VEGF signalling pathway have been hypothesised to induce increased risk of thrombosis due to loss of normal endothelial VEGF receptor-mediated antiplatelet effects which are typically mediated by nitric oxide and prostacyclin [21]. Although each TKI is targeting a different growth factor/pathway, disruption to regular cell function and cytokine production may cause an imbalance of prothrombotic and antithrombotic factors, leading to an RVO. Other classes of small molecule inhibitors, such as MEK inhibitors, have also been linked to RVOs, though these were not part of our cohort and likely act through distinct mechanistic pathways [22]. However, there are not enough studies to confirm or confidently identify a direct mechanism that explains the potential association between TKIs and RVOs.

Patients in our cohort had several common risk factors for RVO, including hypertension, diabetes mellitus, and hyperlipidaemia. Each of these is independently associated with RVO (OR = 3.5 for hypertension, OR = 2.5 for hyperlipidaemia, and OR = 1.5 for diabetes), making it challenging to determine the precise contribution of TKIs to RVO development. While these comorbidities likely contributed substantially to the events we observed, the clustering of RVOs in temporal association with TKI exposure suggests that systemic VEGF inhibition may act as an additional risk factor in predisposed individuals [23]. Additionally, most of these patients were under medical supervision and receiving treatment for their comorbid conditions, suggesting that other factors, including TKI use, may still play a role. The development of RVO is likely multifactorial, with TKIs potentially acting as an additional risk factor, particularly in high-risk individuals. Notably, the majority of patients in our cohort were over the age of 70 (9/11), a well-established risk factor for RVO [24]. Furthermore, most had ocular conditions known to elevate RVO risk, such as glaucoma, NPDR, and hypertensive retinopathy (8/11 patients) [24]. This raises the possibility that in older patients with multiple metabolic comorbidities and ocular manifestations of these diseases, TKIs may heighten the risk of RVO. In contrast, TKI use may not significantly increase RVO risk in individuals who are relatively healthy aside from their indication for the medication. Further studies are needed to assess the impact of TKIs in both high- and lower risk populations to better understand their role in RVO development.

Another important risk factor that should be considered is the patients’ underlying malignancy. Malignancy itself causes hypercoagulability which can subsequently cause RVOs [25]. Despite this theoretical risk, there have been no studies that definitively suggest that there is a higher rate of RVOs in patients with malignancy, compared to the 0.3%-1.6% prevalence rate in the general population [12, 26]. Among patients with colorectal cancer, one study showed an RVO incidence rate of 0.63%, however, it is unclear how this would compare to a control group and whether this is different among other malignancies [27]. Studies have also shown there may be an increased risk of subsequent cancer development among patients who had an RVO, suggesting that there may be a link, but whether cancer increases risk for RVOs has not been fully explored outside of individual case reports [28–34]. In addition, most of the patients in our cohort (8/11) were actually in remission at the time of the RVO and were continuing on their maintenance dose of TKI therapy. Studies have shown that after cancer remission, the risk of thrombosis gradually begins to decrease over time [35]. Since the majority of reported RVO cases occurred during remission, malignancy-related hypercoagulability is less likely to be a confounding factor than it would be if these patients had active cancer. In addition, the temporal association between RVO development and TKI therapy gives credence to a potential role for TKIs in predisposing patients to vascular occlusive events. The likelihood of the RVO being related to TKI use was quantified using the Naranjo Adverse Drug Reaction Scale. The average score was 5.2, indicating a probable association between TKI therapy and RVO development.

In our cohort, only 1 patient experienced bilateral RVOs. The 8 patients who did not discontinue their TKI immediately after the RVO, had a mean follow-up of 2.8 ± 4.4 years while remaining on the medication before being lost to follow-up or discontinuing the drug. It is unclear why these patients did not develop an RVO in the contralateral eye despite continued TKI use. One potential explanation is insufficient follow-up time or changes in TKI dosages after the initial RVO. Of the 8 patients with post-RVO TKI exposure, 2 discontinued the drug after 1 month due to unrelated reasons, 1 had their medication temporarily held for 6 months, and 1 had a dose reduction. It is possible that a longer duration on the prior dose was necessary for a contralateral RVO to develop. However, this does not explain why the remaining 4 patients did not experience an RVO despite extended time on their usual dosing regimen. Another possibility is that intrinsic factors, such as baseline differences in retinal vasculature, collateral circulation, or local inflammatory responses, contributed to the asymmetric development of RVOs. Additionally, systemic factors like fluctuations in blood pressure, haematologic parameters, or other transient prothrombotic conditions may have contributed to the initial RVO, but did not persist long enough to affect the contralateral eye. Given these uncertainties, further investigation is needed to better understand the mechanisms underlying unilateral versus bilateral RVO development in patients on TKIs.

The management of RVO in patients receiving TKI therapy poses unique challenges. Prompt recognition and appropriate treatment are essential to minimise the risk of vision loss and optimise visual outcomes. In our series, intravitreal anti-VEGF therapy was the mainstay of treatment for RVO, with variable improvements in visual acuity observed following intervention. However, decisions regarding the continuation or modification of TKI therapy in the setting of RVO require careful consideration of the underlying malignancy, disease status, and potential risks and benefits of treatment continuation. The additional comorbidities in our study population suggest that patients at particularly high risk for RVO due to systemic and ocular disease may require a reassessment of the optimal therapeutic approach. This is particularly relevant in cases where alternative cancer therapies with similar efficacy are available and may present a lower risk for RVO development. However, there are still no large-scale studies investigating this association, and the clinical decision to alter cancer treatment based on the potential risk of RVO remains difficult for many malignancies. For example, TKIs are the only FDA-approved therapy for patients with GIST. More research is needed to clarify whether TKI discontinuation or modification is warranted in certain high-risk populations. In addition, the safety of initiating a different TKI after an RVO also remains uncertain, and decisions should be made on a case-by-case basis, considering the patient’s risk factors, timing of initiation and the need for ophthalmologic follow-up. At a minimum, patients initiated on TKIs should be informed to be vigilant for vision changes and know how to get into ophthalmology quickly to enable early detection, timely intervention, and potential vision-sparing treatments. A baseline ophthalmic evaluation may be especially prudent for older patients with multiple vascular or ocular risk factors who are initiating VEGF-pathway TKI therapy.

There are several limitations seen in our series. Primarily, the study’s retrospective design prevents us from establishing any causal relationships between TKI use and RVOs. In addition, the small sample size limits statistical power to calculate the relative risk of TKI use and determine statistical significance. The sample size also limits our ability to control for risk factors and comorbid conditions, making it difficult to isolate the impact of TKI use. Lastly, the Naranjo Adverse Drug Reaction Scale has important limitations in the context of vascular occlusions, which are typically multifactorial and for which drug rechallenge is rarely feasible. Consequently, Naranjo scores may overestimate the probability of a causal relationship in this setting, and should be interpreted cautiously alongside clinical context and temporal associations rather than as definitive evidence of causality.

Overall, this series of RVOs occurring during TKI therapy highlights the need for vigilant patient education. Patients on TKIs, particularly those with factors likely to put them at higher risk, should be counselled on the warning signs of an RVO, which should prompt them to see a retina specialist immediately to decrease risk of permanent vision loss.

### What was known before:

- Tyrosine kinase inhibitors (TKIs) are widely used for the treatment of various malignancies.
- Retinal vein occlusion (RVO) has been reported rarely in patients receiving TKIs, but the association remains poorly understood.

### What this study adds:

- We present the largest series to date of RVOs occurring during TKI therapy.
- A temporal relationship and Naranjo scores support a potential causal link between TKIs and RVO.
- This series highlights the importance of ophthalmic monitoring and patient education for those receiving TKIs, particularly individuals at elevated vascular risk.
